# Insights from a 6‐year hair drug analysis compendium in drug‐facilitated sexual assault cases

**DOI:** 10.1111/1556-4029.70331

**Published:** 2026-04-14

**Authors:** Amandine Fort, Coralie Boudin, Hélène Eysseric‐Guerin, Virginie Scolan, Jérémy Borges, François Chiron, Pierre Henry, Françoise Stanke‐Labesque, Théo Willeman

**Affiliations:** ^1^ Clinique de Médecine Légale, CHU Grenoble Alpes Grenoble France; ^2^ Département de médecine légale Univ. Grenoble Alpes Grenoble France; ^3^ Laboratoire de Pharmacologie, Pharmacogénétique et Toxicologie CHU Grenoble Alpes Grenoble France; ^4^ Inserm 1300‐HP2, Univ. Grenoble Alpes Grenoble France

**Keywords:** DFC, DFSA, drug‐facilitated crime, drug‐facilitated sexual assault, forensic toxicology, gamma‐hydroxybutyrate, GHB, hair analysis, mass spectrometry, MS, segmental analysis

## Abstract

Drug‐facilitated sexual assault (DFSA) may involve a diverse array of substances, including illicit drugs, prescription medications, and over‐the‐counter pharmaceuticals. Hair analysis has emerged as a valuable forensic tool, offering an extended window of detection spanning several months. However, interpreting drug concentrations in hair can be challenging in forensic cases, as there are still substantial disparities in drug concentration findings across studies, or even no data available in the literature. This compendium seeks to contribute to the understanding and interpretation of forensic cases involving hair analysis. This study included hair analysis results upon prosecutor request over 6 years in Grenoble Forensic Laboratory from 2019 to 2024. Segmental hair analysis was performed using liquid chromatography coupled to tandem mass spectrometry (LC–MS/MS) on Sciex® 5500QT and Waters® TQ‐XS mass spectrometers, following Society of Hair Testing guidelines. Screened substances included drugs of abuse, benzodiazepines, sedative medications, and gamma‐hydroxybutyrate (GHB), depending on the case, following French Society of Analytical Toxicology guidelines. Hair proficiency quality testing ensured reproducible results. In this compendium, 22 authentic DFSA cases are described with hair analysis. The cohort predominantly involved female victims (95%) aged 13–47 years. Hair analysis was positive in nine cases (41%), revealing the presence of at least one psychoactive substance. Substances identified were alimemazine, alprazolam, bromazepam, cetirizine, clozapine, codeine, cyamemazine, hydroxyzine, oxazepam, zolpidem, and 3,4‐methylenedioxymethamphetamine (MDMA). The chemical profile observed primarily included sedating and amnesic pharmaceuticals, but not only. This compendium adds valuable data in the literature for better hair drug concentration interpretation in forensic cases.

**Highlights**

- Authentic hair analyses contextualized for forensic interpretation.
- Victims were predominantly young adult females.
- Victims' medical treatment must be considered in result interpretation.
- Sedative and amnesic drugs predominated in detected chemical profiles.
- Study enhances forensic interpretation of hair drug concentrations.

## 1 INTRODUCTION

Criminal acts (e.g., sexual assault, homicide) or tortious acts (e.g., robbery, maltreatment) committed against individuals under the influence of psychoactive substances (PAS) are referred to as drug‐facilitated crimes (DFCs). Among these, drug‐facilitated sexual assault (DFSA) is the most prevalent and describes incidents in which the victim is incapacitated and/or unable to provide informed consent as a result of drug or alcohol consumption [1]. Gee et al. [2] further distinguished between proactive DFSA, in which the assailant covertly or forcibly administers an incapacitating or disinhibiting substance to facilitate the assault, and opportunistic DFSA, in which the perpetrator exploits a victim's self‐induced intoxication, often reaching a state of near or complete unconsciousness. Alarmingly, a 2022 survey conducted by the Paris Addictovigilance Center reported a 3% annual increase in suspected chemical submission cases [3], underscoring the growing concern surrounding these offenses.

DFSA involve a wide range of substances, including illicit drugs, prescription medications, and over‐the‐counter drugs. Perpetrators tend to select substances sharing several characteristics: short duration of action, rapid onset of sedation and anterograde amnesia, and short biological detection windows, all of which complicating toxicological confirmation. Although numerous substances may be involved, such as benzodiazepines, related hypnotics (e.g., zopiclone and zolpidem), certain anesthetics, drugs of abuse including cannabis and amphetamine‐type stimulants, and gamma‐hydroxybutyrate (GHB) [4], alcohol remains the most commonly implicated substance in DFSA and is often regarded as the primary “date‐rape” drug [5]. The detection of many of these agents is highly time‐dependent: blood samples typically allow detection over only a few hours, whereas urine samples extended detection to several days. Consequently, delayed reporting or prolonged intervals between the assault and sample collection often limit the effectiveness of conventional toxicological investigations.

Hair analysis has emerged as a valuable forensic tool by extending the detection window for xenobiotic exposure to several months, depending on the length of the hair segment analyzed [6]. This approach is of particular relevance in DFSA investigations, where delayed reporting is common and conventional biological matrices such as blood and urine often fall outside their respective detection windows [7]. Under such circumstances, hair is often the only biological matrix capable of documenting prior substance exposure, thereby providing a retrospective insight that could not be achieved using traditional toxicological analyses. However, hair analysis also presents limitations, including potential external contamination, low analyte concentrations, variability in drug incorporation and hair growth rates, the impact of cosmetic treatments, and challenges in the interpretation of results. These limitations underscore the necessity for rigorous sample collection procedures, the application of appropriate analytical methods, and cautious interpretation of the findings in conjunction with other toxicological and investigative information. Furthermore, data for interpreting drug concentrations in hair are sometimes absent from the scientific literature.

In this context, the present study aims to compile and analyze cases of DFSA through hair analysis at Grenoble Alpes University Hospital in France from 2019 to 2024. This compendium seeks to contribute to the understanding and interpretation of forensic cases involving hair analysis.

## 2 MATERIALS AND METHODS

### 2.1 Case inclusion

All hair samples analyzed were derived from cases officially reported as suspected DFSA, where hair collection and subsequent toxicological analysis were explicitly upon prosecutor's request. Cases were included over 6 years from January 2019 through December 2024.

### 2.2 Hair collection

Hair collection was performed by a trained professional (a toxicologist or a forensic pathologist), at least 4–6 weeks after potential exposure. This interval allowed sufficient time for the hair segment formed during the exposure period to emerge from the scalp, given an average hair growth rate of approximately 1.0 cm per month, thereby enabling detection of incorporated substances in the proximal segment [8]. Sample collection was conducted in accordance with established good practice guidelines [9]. A lock of hair was isolated using a string and cut as close to the scalp as possible from the vertex posterior region, using scissors previously cleaned with an alcohol‐free wipe. The collected lock was secured to a sheet of white paper by taping both ends of the string, ensuring that the adhesive did not come into contact with the hair. The proximal (root) end was clearly indicated. The hair sample was then placed in a labeled envelope, before being shipped to the Grenoble Alpes University Hospital (CHUGA) Pharmacology, Pharmacogenetics and Toxicology Laboratory. Samples were stored at room temperature upon analysis.

### 2.3 Hair strand sample preparation

Each lock of hair was described with the following parameters: color, total length and weight with and without the string. The orientation of hair samples was preserved thanks to the string at the proximal end of the lock of hair. External decontamination was performed by washing the hair samples twice in dichloromethane. In each step, samples were placed in a beaker and manually agitated for 1 min before being blotted dry with absorbent paper. Hair alignment was verified prior to and following the decontamination procedure. The portion to be analyzed was cut into several segments when it was possible. Segments were then finely chiseled into pieces of less than 2 mm and homogenized. Analyses were carried out on test samples ranging from 10 to 20 mg; concentrations were normalized based on the ratio between the actual hair mass analyzed and the mass used for the calibration standards. Sample preparation was identical for all analytical methods and has been previously described in detail [10]. 20 mg of hair were fortified with 10 μL of internal standard at 0.1 mg/L for psychotropic medication, 4 μL at 1.0 mg/L for amphetamines, opiates, cocaine (AOC), and 4 μL at 0.1 mg/L for cannabinoids. After the addition of 400 μL of MeOH, samples were placed in an ultrasonic wash for 10 min and then incubated overnight at 55°C for AOC and cannabinoids, and at ambient temperature for psychotropic medication. Following centrifugation for 20 min at 25,000 rpm, the supernatant was evaporated to dryness under a gentle stream of nitrogen and reconstituted with 200 μL of mobile phase A prior to chromatographic analyses.

For GHB determination, 10 mg of hair were fortified with 5 μL of internal standard (GHB‐d6) at 10 mg/L and incubated at 90°C with 0.5 mL of 1 M NaOH during 20 min. Following incubation, the samples were neutralized with 200 μL of 1 M H2SO4. After the addition of 3 mL ethyl acetate, the mixture was immediately vortexed for 1 min and then placed on a gentle shaker for two 2.5‐min cycles, with manual inversion between cycles. The phases were separated by centrifugation at 3500 rpm for 10 min. The organic layer was then transferred to a borosilicate glass tube and evaporated to dryness under a gentle stream of nitrogen. The dry residue was reconstituted in 40 μL of BSTFA containing 1% TMCS and derivatized in an oven at 65°C for 30 min.

### 2.4 Analytical toxicology procedures

Following the French Society of Analytical Toxicology guidelines [11], several analytical methods were carried out depending on the context and prosecutor's request (Table 1). Both the hair extracts and the decontamination washes were analyzed to assess any possible external contamination.

**TABLE 1:** Scope of toxicological analyses performed according to judicial requisitions.

Case	Matrice	BZD and related products	Sedative psychotropic medication	AOC and metabolites	Cannabinoid	GHB	NPS
1	Hair	Yes (JR)	Yes (JR)	Yes (NR)	No	No	No
2	Hair	Yes (JR)	Yes (JR)	Yes (JR)	Yes (JR)	No	No
3	Hair	Yes (JR)	Yes (JR)	Yes (JR)	Yes (JR)	No	No
4	Hair	Yes (JR)	Yes (JR)	No	No	No	No
	Blood and urine	Yes (JR)	Yes (JR)	Yes (JR)	Yes (JR)	Yes (JR)	Yes (JR)
5	Hair	Yes (JR)	Yes (JR)	No	No	No	No
6	Hair	Yes (JR)	Yes (JR)	Yes (JR)	No	No	No
	Blood	Yes (JR)	Yes (JR)	Yes (JR)	Yes (JR)	Yes (JR)	Yes (JR)
7	Hair	Yes (JR)	Yes (JR)	Yes (JR)	Yes (JR)	No	No
8	Hair	Yes (JR)	Yes (JR)	Yes (JR)	No	No	No
	Blood	Yes (JR)	Yes (JR)	Yes (JR)	Yes (JR)	Yes (JR)	Yes (JR)
9	Hair	Yes (NR)	Yes (NR)	Yes (JR)	Yes (JR)	No	No

*Note*: Yes (JR): analysis performed following a judicial request; Yes (NR): analysis performed without a judicial request; No: analysis not performed.

Abbreviations: AOC, amphetamines, opiates, cocaine; BZD, benzodiazepines; GBH, gamma‐hydroxybutyrate; NPS, new psychoactive substances.

#### 2.4.1 Quantitative LC–MS/MS methods for illicit and sedative drugs

Four quantitative methods were carried out on the different segments.

Firstly, a targeted method of benzodiazepines and related products (29 anxiolytics and/or hypnotic molecules) and secondly a targeted method of sedative psychotropic medication (24 molecules neuroleptics and/or antihistamines) were performed. A methanolic extract obtained after incubating the segments for 12 h in methanol at room temperature was analyzed using LC–MS/MS on a Kinetex® column (XB‐C18 100 Å; 50 × 2.1 mm; Phenomenex, 2.6 μm). A comprehensive description of the chromatographic conditions and MS parameters is provided in Table S1.

Then, a method for AOC and metabolites and lastly a method for cannabinoids were performed, as previously described [12], on a LC system (Shimadzu, Kyoto, Japan) consisting of two LC‐20AD quaternary pumps and two LC‐20AD XR quaternary pumps equipped with a SIL‐20AC XR autosampler and a CTO‐20AC column compartment. Cannabinoids separation was performed with an online sample purification on a pentafluorophenyl (PFP) Kinetex column 5 μm × 2.1 mm × 50 mm (Phenomenex, Aschaffenburg, Germany) and a chromatographic separation was performed on an XB C18 analytical column Kinetex 5 μm × 2.1 mm × 50 mm (Phenomenex, Aschaffenburg, Germany). AOC and metabolites separation were managed on a single biphenyl column Kinetex 3 μm × 2.1 mm × 100 mm (Phenomenex, Aschaffenburg, Germany).

Detection for the four methods was performed on an API 5500 tandem mass spectrometer (Sciex, Toronto, Canada) equipped with a Turbo Ion Spray® source. Quantification was achieved in the multiple reaction monitoring (MRM) mode, with one transition for quantification, one transition for confirmation per analyte, and one ion transition per internal standard.

#### 2.4.2 Quantitative GC–MS methods for GHB

GHB determination was performed on the different segments by GC/MS after basic hydrolysis, liquid–liquid extraction and silylation on an Agilent Technologies system combining a 7890D Network GC System with a 5977‐network mass selective detector equipped with a high‐efficiency source. Samples were injected onto a DB‐5 MS UI column (30 m–0.25 mm internal diameter; 0.25 μm film thickness) by pulsed split‐less injection at an injector temperature of 250°C. Quantification was achieved in the selected ion monitoring (SIM) mode. For this purpose, smaller hair segments of 5 mm were analyzed to properly discriminate exogenous exposure.

#### 2.4.3 Screening LC–MS/MS methods for medications and NPS

Two qualitative screening methods were also performed as already described [13]. The acquisition consisted of 2 scheduled targeted screenings containing 349 drugs and 166 new psychoactive substances (NPS), respectively. Ultrahigh performance liquid chromatography (UPLC) was performed on an I‐class Acquity system (Waters Milford, USA). Chromatographic separation was achieved using an Acquity HSS T3 column (100 mm × 2.1 mm, 2.5 μm) (Waters). Qualitative targeted screening was performed on a Xevo TQ‐XS (Waters).

#### 2.4.4 Methods validation

A blank hair sample was tested in each batch to ensure no contamination has occurred.

Internal quality controls (IQC) were conducted for a series of assays, encompassing both commercial IQCs specific to our laboratory and three in‐house IQCs using spiked drug‐free head hair.

Accuracy, precision, lower limits detection (LOD) and lower limits of quantification (LOQ) are reported in Table S2.

Hair proficiency quality testing ensured reproductive results, with programs such as Arvecon—DHF or French Society of Analytical Toxicology—SOUCHI yielding satisfactory results.

### 2.5 Ethics

This study concerns forensic toxicological analysis at the request of the law enforcement authorities or a magistrate. Because this study used only routinely collected, de‐identified data, it did not require any ethics committee approval, and informed consent was waived in accordance with French regulations for mandatory reporting by health care professionals (Article R5132‐102 of the Public Health Code).

## 3 RESULTS

### 3.1 Population description

**FIGURE 1:**
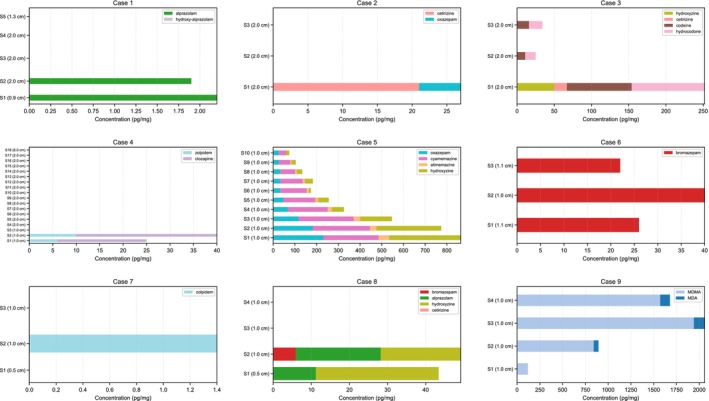
Segmental hair toxicological findings in DFSA cases. Drug concentrations (pg/mg) detected in successive hair segments for each individual case. Segment lengths are indicated in parentheses.

Between 2019 and 2022, a total of 22 DFSA cases were identified. The mean age of the victims was 25 years, and the majority were women. The demographic characteristics of the study population are presented in Table 2. Hair toxicological analyses were performed in all cases. Hair samples were segmented, with mean segment lengths ranging from 0.9 to 2 cm (Figure 1).

**TABLE 2:** Population characteristics.

Variable (*N* = 22)		Percentage (%*N*)
Sociodemographic		
Mean age (min–max) (years)	25 (13–47)	
Sex		
Female, *n*	21	95
Male, *n*	1	5
Positives cases, *n*	9	41
Victims' contexts		
Location		
Home, *n*	7	44
Private place, *n*	6	38
Public place, *n*	3	19
*Missing data*	*6*	
Reported symptoms		
Amnesia, *n*	9	41
Vomiting, *n*	4	18
Dizziness, *n*	3	14
Nausea, *n*	3	14
Hypotonia, *n*	2	9
Drowsiness, *n*	2	9
Fainting spell, *n*	1	5
Memory problems, *n*	1	5
Headache, *n*	1	5
Confusion, *n*	1	5
Disorientation, *n*	1	5
Asthenia, *n*	1	5
*Missing data*	*9*	
Hair samples characteristics		
Hair type		
Scalp, *n*	22	100
Mean sample's weight (min‐max) (mg)	680 (129–1761)	
Hair color		
Brown, *n*	16	73
Dark, *n*	4	18
Light, *n*	2	9
Hair treatment		
Participants without hair bleaching, *n*	15	68
Participant with hair bleaching, *n*	7	32

Nine DFSA cases (41% of the cases identified during the study period), including one case related to robbery, showed the presence of substances in hair samples (Table 3; Figure 1). In three of these positive cases, urine and blood analyses were also available, with biological sample collection occurring between 20 and 25 h after the alleged exposure.

**TABLE 3:** Demographic characteristics, clinical presentation, and toxicological findings in DFSA cases.

Case	Sex	Age (years)	Suspected vector	Symptoms	Hair analysis				Substance	Hair concentrations from root to tip (pg/mg)	Other positive matrices	
					Time between exposure and hair sampling (days)	Color	Hair bleaching	Number of analyzed segments			Blood (ng/mL)	Urine (ng/mL)
1	F	34	Unknown	Unknown	49	Brown	Yes	5	Alprazolam^a^	2.2/1.9^b^/ND/ND/ND	na	na
									Hydroxy‐alprazolam	Traces/Traces^b^/ND/ND/ND		
2	F	19	Unknown	Drowsiness	49	Dark	No	3	Cetirizine	21^b^/ND/ND	na	na
				Hypotonia					Oxazepam^a^	6/ND/ND		
3	F	19	Unknown	Drowsiness	49	Brown	No	2	Hydroxyzine	50^b^/ND/ND	na	na
				Hypotonia					Cetirizine	17^b^/ND/ND		
									Codeine	87^b^/11/16		
									Hydrocodone	98^b^/14/18		
4	F	37	Drink	Unknown	42	Brown	Yes	18	Zolpidem	6/10^b^/ND in all following segments	Zolpidem (0.3)	Zolpidem (1.0)
									Clozapine	19/30^b^/ND in all following segments	Clozapine (4.8)	Clozapine (6.2)
											Norclozapine (2)	Norclozapine (178)
5	F	30	Pill	Unknown	90	Brown	Yes	10	Oxazepam^a^	231/182/117^b^/68/47/32/32/31/26/25	na	na
									Cyamemazine	255/264/254^b^/183/147/122/104/70/53/34		
									Alimemazine	48/30/30^b^/19/13/10/10/8/6/ND		
									Hydroxyzine	330/298/146^b^/56/49/10/37/25/19/15		
6	F	31	Unknown	Dizziness	120	Dark	No	2	Bromazepam	26/40/22^b^	Bromazepam (177)	na
				Nausea							OH‐bromazépam (36)	
				Confusion								
				Disorientation								
7	M	22	Drink	Drowsiness	30	Brown	No	4	Zolpidem	ND/1.4^b^/ND	na	na
8	F	19	Drink	Amnesia	60	Brown	No	4	Bromazepam	ND/5.9^b^/ND^b^/ND	ND	na
				Vomiting					Alprazolam^a^	11.2/22.3^b^/ND^b^/ND		
									Hydroxyzine^a^	32.2/21^b^/ND^b^/ND		
									Cetirizine	nq/nq^b^/ND^b^/ND		
9	F	13	Unknown	Unknown	60	Dark	Yes	4	MDMA	118/841^b^/1941/1571	na	na
									MDA	ND/53^b^/118/107		

Abbreviations: na, not analyzed; ND, not detected; nq, not quantified.

^a^ Victim's treatment.

^b^ Segment corresponding to alleged facts.

The scope of toxicological analyses varied between cases, depending on the content of the judicial requisitions (Table 1). Specific analytical methods for GHB and screening procedures for NPS were performed in urine and blood samples in three positive cases but all results for GHB and NPS in these matrices were negative. In addition, specific analyses for GHB were conducted in hair samples from seven cases classified as negative, and NPS screening in hair was performed in one case; all of these analyses yielded negative results.

Excluding prescribed medications, the most frequently detected substances were cetirizine (*n* = 3), hydroxyzine (*n* = 2), zolpidem (*n* = 2), and bromazepam (*n* = 2). In three cases, a beverage was identified as the suspected vector for drug administration.

### 3.2 Forensic cases description

Case 1: A 34‐year‐old female with brown, bleached hair reported being on a prescribed treatment of alprazolam (0.25 mg/day) and dosulepine (25 mg/day). Limited contextual information was available for this case.

Case 2 A 19‐year‐old female with dark hair reported a coercive sexual act after experiencing drowsiness and muscular hypotonia during an evening and night spent with a man. An anxiolytic treatment (oxazepam, 5 mg/day) was initiated 15 days after the alleged incident.

Case 3: A 19‐year‐old female with brown hair experienced sudden drowsiness and muscular hypotonia while at a party in a private residence. She awoke to non‐consensual sexual activity.

Case 4: A 37‐year‐old female with bleached brown hair reported non‐consensual sexual acts after consuming a cup of milk with an unusual taste. Trace amounts of zolpidem (1.27 ng/mL) and clozapine (45.7 ng/mL) were detected in the milk bottle and her urine and blood samples.

Case 5: A 30‐year‐old female with bleached brown hair reported that an individual forcibly administered 20 tablets of her prescribed oxazepam (Seresta®, 50 mg), a medication she typically took at a daily dose of half a tablet. She subsequently reported non‐consensual sexual intercourse.

Case 6: A 31‐year‐old female with dark hair experienced malaise, nausea, dizziness and altered alertness after lunch with an individual who attempted to kiss her. She reported no ongoing medical treatment but used CBD oil for back pain. Bromazepam (177 ng/mL) was detected in her blood sample.

Case 7: A 22‐year‐old male with brown hair presented with drowsiness after ingesting an undisclosed substance provided by a third party. He also reported voluntary alcohol consumption beforehand. He subsequently reported experiencing non‐consensual sexual contact during the night.

Case 8: A 19‐year‐old female with brown hair reported emesis and anterograde amnesia after consuming ethanol and a taurine‐containing energy drink. She reported being the victim of a non‐consensual sexual act. Her documented pharmacological history included hydroxyzine and alprazolam.

Case 9: A 13‐year‐old female reported the unwitting ingestion of MDMA, followed by a non‐consensual sexual act. She had a prior instance of MDMA consumption within the preceding month.

## 4 DISCUSSION

The demographic characteristics of our study population, primarily female with a mean age of 25 years, were consistent with existing literature and a prior French cohort [5, 14]. It should be noted that some studies, such as one conducted in Eastern Denmark, suggested a potential underreporting of sexual assault among male victims, possibly due to a lower likelihood of reporting incidents to law enforcement compared with female victims [15]. In cases of sexual assaults, evidentiary elements are often limited, particularly when the victims were not examined promptly after the alleged events. When chemical submission is suspected, the detection of substances becomes crucial for the investigation, lending significant weight to the victim's testimony. Recognizing this, Liautard et al. recommended the systematic implementation of blood and urine toxicological analyses in all suspected or proven DFC [16]. In the present study, the limited availability of blood and urine samples, obtained in only five cases, reflected delays in victim reporting and subsequent sample collection.

Toxicological analyses were not performed in a systematic manner across cases. In most instances, judicial requisitions provided little or no specification regarding the analytical toxicology procedures, often restricting the request to the non‐specific mandate of “detecting any substance that could be used in the context of drug‐facilitated crime”. Notably, in cases classified as positive, GHB and NPS were rarely investigated. When blood and urine samples were available, analyses were preferentially conducted on these matrices, and analyses were not subsequently performed on hair samples. In other cases, the analytical strategy appeared to have been oriented according to clinical and anamnestic information, particularly the symptoms reported by the victim.

The toxicological findings of the present study were consistent with those reported in other European studies, which frequently identified drugs such as benzodiazepines and analgesics [14, 17, 18, 19]. However, antihistamines were detected more frequently in the present cohort than in previously published studies. Alcohol was not detected in the two cases where both urine and blood samples were available. This observation contrasted with findings from several studies that identified ethanol as the most frequently implicated substance in sexual assault cases [5]. Interestingly, a 2019 Australian study also highlighted the low detection rate of alcohol despite it being the most commonly reported substance consumed in such contexts [20].

Although our targeted analytical method included 166 NPS (including cathinones identified in DFSA cases) as recommended by the French Society of Analytical Toxicology [21], none were detected in any of our cases when performed. This finding contrasted with reports such as Larabi et al., who described a DFSA case involving 4‐methylethcathinone (4‐MEC), 3,4‐methylenedioxypyrovalerone (MDPV), and doxylamine [22]. Furthermore, no DFSA cases were identified within a “chemsex” context in the present study, despite such cases having been reported in France during the same period [23].

Our observations also raised important questions regarding the origin of the detected substances. The presence of both over‐the‐counter and prescription medications prompts consideration as to whether these substances are part of the legitimate medical treatments in the individuals concerned, potentially reflecting underlying psychiatric or medical conditions that may warrant further investigation. Alternatively, these substances may have been obtained through illicit channels.

In France, the regulatory framework governing codeine has been progressively reinforced. Since July 2017, codeine‐containing medicines are no longer available over the counter and require a medical prescription, with dispensing subject to stricter controls. Furthermore, since March 2025, codeine prescriptions in specific situations are required to be issued on secured prescription forms, in accordance with national regulations aimed at reducing misuse, diversion, and dependence [24]. This regulatory evolution offers a unique opportunity for future research to monitor potential shifts in the prevalence of codeine detection in hair samples and to evaluate the effectiveness of such measures. Similar considerations have previously been raised in the context of tetrahydrozoline misuse [25].

The most frequently reported clinical manifestations in confirmed positive cases were drowsiness (*n* = 3) and hypotonia (*n* = 2). Notably, the two cases presenting with both drowsiness and hypotonia showed the presence of hydroxyzine, whereas zolpidem was identified in the remaining case reporting drowsiness. These clinical findings were consistent with the established pharmacological profiles of these substances [26, 27, 28]. According to the summaries of product characteristics available in the public drug database, drowsiness is reported as an adverse effect in 9.63% of adult patients taking cetirizine and in 13.74% of those taking hydroxyzine. Combination use with other central nervous system depressants, as observed in cases 2, 3, 5, and 8, may have increased the sedative effects induced by antihistamines. The symptoms reported in these cases were therefore in line with the known pharmacological profiles of the identified compounds. Furthermore, in cases where multiple biological matrices were available, the suspected substances were consistently detected across all samples. This consistency strengthened the credibility of the findings and effectively ruled out environmental contamination.

When comparing the concentrations observed in the present study with those reported by Xiang et al. [28] in a comprehensive literature review of confirmed DFSA cases, similarities were observed for certain compounds in hair. For instance, the concentrations measured were comparable to those previously reported following a 100 mg unit dose of codeine (up to 570 pg/mg vs. 87 pg/mg in Case 3), and a 6 mg unit dose of bromazepam (up to 28 pg/mg vs. 40 pg/mg in Case 6). In contrast, oxazepam concentrations were markedly lower than those reported by Kintz et al. [29], despite an alleged higher unit dose in the present study (50 mg vs. 10 mg). This discrepancy was particularly evident in Case 2, in which oxazepam was administered as part of a therapeutic regimen.

Hydroxyzine concentrations were much higher than those previously reported following a 25 mg tablet, with the exception of Case 8, in which hydroxyzine was part of the patient's treatment. Similarly, alimemazine concentrations exceed those reported for a 5 mg unit dose (7 pg/mg vs. 30 pg/mg in Case 5), and cyamemazine concentrations were considerably higher than published values, even in the context of DFC (37–66 pg/mg vs. >250 pg/mg in Case 5). Zolpidem concentrations exhibited interindividual variability, ranging from 1.2 to 555 pg/mg for comparable unit doses, thereby largely confirming the approximately 10‐fold variation observed in two cases of the present study. Comparable variability has also been described for clozapine, for which hair concentrations appear to be more strongly influenced by the administered dose [30].

These substantial discrepancies and the high variability observed in hair concentrations strongly underscored the need for cautious interpretation of toxicological findings, especially concerning the timing of drug intake when the detected substances are part of the victim's treatment regimen [31].

The use of stimulants, particularly MDMA, in proactive DFSA has been increasingly reported [3, 11]. In the present study, the MDMA concentrations measured in Case 9 were much higher than those reported in other confirmed DFSA cases [22, 32]. This case illustrated an important limitation of hair analysis in distinguishing a single, unintentional exposure from chronic use of this substance. Undeclared voluntary consumption by the victim could not be excluded. Although changes in patterns of use may be detectable in cases of increased or repeated intake, a single exposure may not be reliably differentiated from chronic use based solely on hair analysis.

GHB was quantified in seven cases, which were classified as endogenous findings rather than positive results for exogenous administration. GHB was detected in all analyzed hair segments across these cases, with concentrations ranging from 0.31 to 5.03 ng/mg. The maximum concentration ratio observed between any two segments within a single case was a factor of five. According to the United Nations Office on Drugs and Crime (UNODC) Guidelines for the Forensic Analysis of Drugs Facilitating Sexual Assault and Other Criminal Acts, a concentration differential of at least 10 times higher in one segment compared with others is necessary to strongly suggest exogenous exposure. Consistent with prior research [33, 34], neither the absolute GHB concentrations nor the segmental variations observed in our study met this criterion for exogenous administration.

Among the analyzed cases, 13 were negative for the presence of commonly implicated DFSA substances. In these negative hair cases, the median interval between the alleged assault and hair sample collection was 60 days (range: 49–730 days). Only three of these cases involved bleached hair, and only one involved light hair. These observations suggest that external factors such as weathering, environmental pollution, or cosmetic treatments might contribute to the degradation and reduced detectability of drugs in hair [35]. In this group of negative hair cases, alternative biological matrices were available in only one case, in which ethanol and acetaminophen were detected in blood, and ethanol, acetaminophen, and ibuprofen were detected in urine.

It is important to emphasize that the concentrations reported in the present study represent comparative values within a confirmed DFSA context and should not be considered definitive reference standards. Individual variability and differences in laboratory preparation and analytical procedures contribute to discrepancies in measured concentrations. Furthermore, the minimum detectable dose in hair remains unknown, and detection thresholds may vary between laboratories. Consequently, a negative hair result does not formally exclude involuntary substance administration [36].

## 5 CONCLUSION

This study provides a comprehensive compendium of DFSA cases, detailing victim age ranges, hair properties, and relevant timeframes. This data offers critical comparative insights for interpreting findings in living victims and enhances our understanding of dose–response relationships in this population. The wide variety of molecules identified and their diverse medico‐legal implications underscore the need for continued reporting and enrichment of cases in the scientific literature.

## CONFLICT OF INTEREST STATEMENT

The authors have no conflicts of interest to declare.

## Supporting information


Table S1.

Table S2.

## Data Availability

The data that support the findings of this study are available from the corresponding author upon reasonable request.
