# Supplementary material for: Insights from a 6‐year hair drug analysis compendium in drug‐facilitated sexual assault cases
Source: J Forensic Sci. 2026 Apr 14;71(4):1783–92. doi: 10.1111/1556-4029.70331 (PMC13340955; doi:10.1111/1556-4029.70331)
Supplement: Supplementary file 1 — Table S1. [file JFO-71-1783-s002.docx]

TABLE S1 MRM transitions, voltage settings declustering potential (DP), entrance potential (EP), collision energy (CE), and collision cell exit potential (CXP) for each analyte of the MS/MS methods on API5500 QTRAP mass spectrometer.

The source parameters was set as follows: electrospray positive voltage: 5500 V, Ion source temperature: 500°C; collision gas (CAD): 6 psi; Curtain Gas 30 psi; nebulization gas: 45 psi; turbo heater gas: 60 psi, Entrence Potential (EP): 10 V. Dwell times were set automatically by the Scheduled MRM mode (target scan time fixed time 0.5 sec). MRM transitions noted “1” were used as quantifiers.

| **AOC method** | **Q1 m/z)** | **Q3 (m/z)** | **DP** | **CE** | **CXP** |
| --- | --- | --- | --- | --- | --- |
| Amphetamine 1 | 136.000 | 119.000 | 36.00 | 13.00 | 12.00 |
| Amphetamine 2 | 136.000 | 91.000 | 36.00 | 25.00 | 8.00 |
| Metamphetamine 1 | 150.000 | 119.000 | 51.00 | 15.00 | 10.00 |
| Metamphetamine 2 | 150.000 | 65.000 | 51.00 | 49.00 | 6.00 |
| Mephedrone 1 | 178.100 | 145.000 | 41.00 | 27.00 | 12.00 |
| Mephedrone 2 | 178.100 | 144.000 | 41.00 | 41.00 | 10.00 |
| MDA 1 | 180.000 | 135.000 | 29.00 | 25.00 | 10.00 |
| MDA 2 | 180.000 | 133.000 | 29.00 | 25.00 | 10.00 |
| MDMA 1 | 194.000 | 105.000 | 35.00 | 31.00 | 8.00 |
| MDMA 2 | 194.000 | 163.000 | 35.00 | 17.00 | 12.00 |
| EME 1 | 200.100 | 182.100 | 26.00 | 21.00 | 6.00 |
| EME 2 | 200.100 | 82.100 | 26.00 | 31.00 | 6.00 |
| MDEA 1 | 208.000 | 105.000 | 27.00 | 33.00 | 8.00 |
| MDEA 2 | 208.000 | 163.000 | 27.00 | 17.00 | 12.00 |
| EDDP 1 | 278.100 | 234.100 | 80.00 | 50.00 | 12.00 |
| EDDP 2 | 278.100 | 186.000 | 80.00 | 46.00 | 10.00 |
| Norcodeine 1 | 286.100 | 152.000 | 80.00 | 75.00 | 10.00 |
| Norcodeine 2 | 286.100 | 165.000 | 80.00 | 58.00 | 10.00 |
| Morphine 1 | 286.200 | 152.000 | 91.00 | 73.00 | 10.00 |
| Morphine 2 | 286.200 | 128.000 | 91.00 | 73.00 | 10.00 |
| BZE 1 | 290.200 | 168.000 | 60.00 | 35.00 | 12.00 |
| BZE 2 | 290.200 | 105.000 | 60.00 | 50.00 | 10.00 |
| Hydrocodone 1 | 300.100 | 199.000 | 100.00 | 50.00 | 12.00 |
| Hydrocodone 2 | 300.100 | 128.000 | 100.00 | 70.00 | 10.00 |
| Codeine 1 | 300.100 | 152.000 | 105.00 | 83.00 | 12.00 |
| Codeine 2 | 300.100 | 215.000 | 105.00 | 35.00 | 10.00 |
| Oxymorphone 1 | 302.000 | 227.100 | 86.00 | 39.00 | 18.00 |
| Oxymorphone 2 | 302.000 | 198.100 | 86.00 | 57.00 | 14.00 |
| Dihydrocodeine 1 | 302.100 | 199.100 | 106.00 | 43.00 | 6.00 |
| Dihydrocodeine 2 | 302.100 | 171.100 | 106.00 | 54.00 | 8.00 |
| Noroxycodone 1 | 302.100 | 187.100 | 40.00 | 33.00 | 10.00 |
| Noroxycodone 2 | 302.100 | 227.100 | 40.00 | 40.00 | 10.00 |
| Cocaine 1 | 305.200 | 183.000 | 120.00 | 20.00 | 14.00 |
| Cocaine 2 | 304.200 | 105.000 | 120.00 | 45.00 | 10.00 |
| Ethylmorphine 1 | 314.100 | 152.200 | 86.00 | 89.00 | 10.00 |
| Ethylmorphine 2 | 314.100 | 115.100 | 86.00 | 101.00 | 10.00 |
| Oxycodone 1 | 316.100 | 241.100 | 75.00 | 38.00 | 8.00 |
| Oxycodone 2 | 316.100 | 256.100 | 75.00 | 33.00 | 8.00 |
| Cocaethylene 1 | 319.100 | 197.100 | 61.00 | 35.00 | 12.00 |
| Cocaethylene 2 | 319.100 | 83.000 | 61.00 | 45.00 | 8.00 |
| 6-MAM 1 | 328.200 | 211.200 | 130.00 | 35.00 | 6.00 |
| 6-MAM 2 | 328.200 | 165.200 | 130.00 | 49.00 | 14.00 |
| Pholcodine 1 | 399.200 | 114.000 | 106.00 | 41.00 | 8.00 |
| Pholcodine 2 | 399.200 | 381.000 | 106.00 | 46.00 | 12.00 |
| Methadone 1 | 310.296 | 265.200 | 81.00 | 21.00 | 8.00 |
| Methadone 2 | 310.296 | 105.200 | 81.00 | 39.00 | 8.00 |
| NorBup 1 | 414.209 | 83.300 | 111.00 | 71.00 | 6.00 |
| NorBup 2 | 414.209 | 101.300 | 126.00 | 50.00 | 12.00 |
| NorBup-D3 | 417.267 | 83.200 | 126.00 | 77.00 | 6.00 |
| Bup 1 | 468.250 | 55.300 | 126.00 | 91.00 | 2.00 |
| Bup 2 | 468.250 | 468.250 | 131.00 | 50.00 | 14.00 |
| N-desmethyl-tramadol 1 | 250.109 | 44.200 | 61.00 | 31.00 | 6.00 |
| O-Desmethyl-tramadol 1 | 250.120 | 58.200 | 66.00 | 37.00 | 10.00 |
| O-Desmethyltramadol 2 | 250.200 | 232.100 | 66.00 | 37.00 | 10.00 |
| Tramadol 1 | 264.118 | 58.200 | 66.00 | 45.00 | 10.00 |
| Dextromethorphan 1 | 272.180 | 215.300 | 101.00 | 37.00 | 14.00 |
| Dextromethorphan 2 | 272.180 | 147.100 | 101.00 | 41.00 | 10.00 |
| Fentanyl 1 | 337.230 | 188.200 | 96.00 | 33.00 | 12.00 |
| Fentanyl 2 | 337.230 | 105.100 | 96.00 | 53.00 | 6.00 |

| **Cannabinoids method** | **Q1 m/z)** | **Q3 (m/z)** | **DP** | **CE** | **CXP** |
| --- | --- | --- | --- | --- | --- |
| THC 1 | 315.100 | 193.100 | 141.00 | 29.00 | 14.00 |
| THC 2 | 315.100 | 123.000 | 141.00 | 41.00 | 16.00 |
| THC-OH 1 | 331.100 | 193.000 | 51.00 | 33.00 | 12.00 |
| THC-OH 2 | 331.100 | 313.100 | 51.00 | 33.00 | 19.00 |
| THC-COOH 1 | 345.100 | 193.200 | 100.00 | 37.00 | 10.00 |
| THC-COOH 2 | 345.100 | 299.100 | 100.00 | 25.00 | 20.00 |
| CBD 1 | 315.100 | 193.200 | 100.00 | 30.00 | 12.00 |
| CBD 2 | 315.100 | 259.000 | 100.00 | 25.00 | 12.00 |
| CBD-OH 1 | 331.100 | 193.200 | 51.00 | 33.00 | 12.00 |
| CBD-OH 2 | 331.100 | 313.100 | 51.00 | 33.00 | 19.00 |
| CBD-COOH 1 | 345.100 | 193.210 | 100.00 | 37.00 | 10.00 |
| CBD-COOH 2 | 345.100 | 299.110 | 100.00 | 25.00 | 20.00 |
| HHC 1 | 317.200 | 193.100 | 120.00 | 33.00 | 14.00 |
| HHC 2 | 317.200 | 123.100 | 120.00 | 47.00 | 20.00 |
| HHC-COOH 1 | 347.200 | 193.200 | 150.00 | 39.00 | 12.00 |
| HHC-COOH 2 | 347.200 | 301.100 | 150.00 | 33.00 | 21.00 |

| **Sedatives method** | **Q1 m/z)** | **Q3 (m/z)** | **DP** | **CE** | | **CXP** | |
| --- | --- | --- | --- | --- | --- | --- | --- |
| Norketamine 1 | 224.130 | 125.000 | 66.00 | 35.00 | | 2.00 | |
| Norketamine 2 | 224.130 | 89.100 | 66.00 | 67.00 | | 2.00 | |
| Ketamine 1 | 238.150 | 125.100 | 71.00 | 35.00 | | 4.00 | |
| Ketamine 2 | 238.150 | 179.100 | 71.00 | 21.00 | | 4.00 | |
| Pheniramine 1 | 241.165 | 196.200 | 66.00 | 21.00 | | 2.00 | |
| Pheniramine 2 | 241.165 | 167.100 | 66.00 | 51.00 | | 2.00 | |
| Diphenhydramine 1 | 256.203 | 167.200 | 51.00 | 17.00 | | 4.00 | |
| Diphenhydramine 2 | 256.203 | 152.200 | 51.00 | 49.00 | | 4.00 | |
| Doxylamine 1 | 271.226 | 182.200 | 61.00 | 21.00 | | 4.00 | |
| Doxylamine 2 | 271.226 | 167.200 | 61.00 | 45.00 | | 4.00 | |
| Chlorpheniramine 1 | 275.061 | 230.000 | 66.00 | 21.00 | | 4.00 | |
| Chlorpheniramine 2 | 275.061 | 167.300 | 66.00 | 51.00 | | 4.00 | |
| Prometazine 1 | 285.038 | 86.000 | 66.00 | 23.00 | | 4.00 | |
| Prometazine 2 | 285.038 | 198.000 | 66.00 | 35.00 | | 4.00 | |
| Atropine 1 | 290.142 | 124.200 | 96.00 | 33.00 | | 4.00 | |
| Atropine 2 | 290.142 | 92.900 | 96.00 | 41.00 | | 4.00 | |
| Alimemazine 1 | 299.235 | 100.200 | 81.00 | 23.00 | | 4.00 | |
| Alimemazine 2 | 299.235 | 180.100 | 81.00 | 57.00 | | 4.00 | |
| Trihexyphenidyle 1 | 302.155 | 98.100 | 71.00 | 31.00 | | 4.00 | |
| Trihexyphenidyle 2 | 302.155 | 284.100 | 71.00 | 21.00 | | 4.00 | |
| Scopolamine 1 | 304.222 | 138.200 | 76.00 | 25.00 | | 4.00 | |
| Scopolamine 2 | 304.222 | 156.200 | 76.00 | 21.00 | | 4.00 | |
| Chlorpromazine 1 | 318.999 | 86.200 | 71.00 | 27.00 | | 4.00 | |
| Chlorpromazine 2 | 319.226 | 58.100 | 86.00 | 47.00 | | 4.00 | |
| Brompheniramine 1 | 320.955 | 276.000 | 66.00 | 21.00 | | 4.00 | |
| Brompheniramine 2 | 320.955 | 167.000 | 66.00 | 55.00 | | 4.00 | |
| Cyamemazine 1 | 324.232 | 100.200 | 81.00 | 25.00 | | 4.00 | |
| Cyamemazine 2 | 324.232 | 205.200 | 81.00 | 55.00 | | 4.00 | |
| Levomepromazine 1 | 329.289 | 100.200 | 81.00 | 25.00 | | 4.00 | |
| Levomepromazine 2 | 329.289 | 242.200 | 81.00 | 27.00 | | 4.00 | |
| Paroxetine 1 | 330.173 | 70.100 | 86.00 | 47.00 | | 2.00 | |
| Paroxetine 2 | 330.173 | 192.300 | 86.00 | 23.00 | | 2.00 | |
| Tropatepine 1 | 334.048 | 178.100 | 96.00 | 55.00 | | 4.00 | |
| Tropatepine 2 | 334.048 | 211.200 | 96.00 | 37.00 | | 4.00 | |
| Niaprazine 1 | 357.299 | 106.100 | 86.00 | 51.00 | | 4.00 | |
| Niaprazine 2 | 357.299 | 177.200 | 86.00 | 27.00 | | 4.00 | |
| Hydroxyzine 1 | 375.283 | 201.200 | 71.00 | 25.00 | | 4.00 | |
| Hydroxyzine 2 | 375.283 | 165.100 | 71.00 | 79.00 | | 4.00 | |
| Haloperidol 1 | 376.253 | 123.100 | 86.00 | 55.00 | | 4.00 | |
| Haloperidol 2 | 376.253 | 165.200 | 86.00 | 31.00 | | 4.00 | |
| Loratadine 1 | 383.005 | 337.100 | 96.00 | 27.00 | | 4.00 | |
| Loratadine 2 | 383.005 | 267.200 | 96.00 | 47.00 | | 4.00 | |
| Cetirizine 1 | 389.145 | 201.100 | 66.00 | 23.00 | | 4.00 | |
| Cetirizine 2 | 389.145 | 165.200 | 66.00 | 85.00 | | 4.00 | |
| Risperidone 1 | 411.346 | 191.100 | 101.00 | 41.00 | | 4.00 | |
| Risperidone 2 | 411.346 | 110.200 | 101.00 | 69.00 | | 4.00 | |
| OH Risperidone 1 | 427.110 | 207.200 | 96.00 | 37.00 | | 2.00 | |
| OH Risperidone 2 | 427.110 | 110.100 | 96.00 | 59.00 | | 2.00 | |
| Clozapine 1 | 327.100 | 270.100 | 100.00 | 46.00 | | 4.00 | |
| Clozapine 2 | 327.100 | 192.100 | 100.00 | 46.00 | | 4.00 | |
| **Benzodiazepines method** | **Q1 m/z)** | **Q3 (m/z)** | **DP** | **CE** | **CXP** | |  |
| Nordiazepam 1 | 271.118 | 140.100 | 111.00 | 39.00 | 4.00 | |  |
| Nordiazepam | 271.118 | 165.100 | 111.00 | 39.00 | 4.00 | |  |
| Nitrazepam 1 | 282.187 | 236.200 | 101.00 | 37.00 | 4.00 | |  |
| Nitrazepam | 282.187 | 180.100 | 101.00 | 51.00 | 4.00 | |  |
| Flunitrazepam 7 Amino 1 | 284.204 | 135.200 | 91.00 | 37.00 | 4.00 | |  |
| Flunitrazepam 7 Amino | 284.204 | 227.300 | 91.00 | 35.00 | 4.00 | |  |
| Diazepam | 285.158 | 154.200 | 96.00 | 39.00 | 4.00 | |  |
| Diazepam 1 | 285.158 | 193.200 | 96.00 | 45.00 | 4.00 | |  |
| Clonazepam 7 Amino 1 | 286.135 | 121.100 | 101.00 | 43.00 | 4.00 | |  |
| Clonazepam 7 Amino | 286.135 | 222.200 | 101.00 | 37.00 | 4.00 | |  |
| Oxazepam 1 | 287.116 | 241.000 | 81.00 | 33.00 | 4.00 | |  |
| Oxazepam | 287.116 | 269.200 | 81.00 | 19.00 | 4.00 | |  |
| Clobazam DM | 287.131 | 245.100 | 91.00 | 25.00 | 4.00 | |  |
| Clobazam DM 1 | 287.131 | 210.100 | 91.00 | 45.00 | 4.00 | |  |
| Tetrazepam 1 | 289.138 | 225.200 | 96.00 | 41.00 | 4.00 | |  |
| Tetrazepam | 289.138 | 253.100 | 96.00 | 37.00 | 4.00 | |  |
| Estazolam 1 | 295.222 | 267.100 | 91.00 | 35.00 | 4.00 | |  |
| Estazolam | 295.222 | 205.300 | 91.00 | 53.00 | 4.00 | |  |
| Flunitrazepam DM 1 | 300.186 | 254.100 | 96.00 | 37.00 | 4.00 | |  |
| Flunitrazepam DM | 300.186 | 198.200 | 96.00 | 49.00 | 4.00 | |  |
| Temazepam 1 | 301.157 | 255.200 | 81.00 | 29.00 | 4.00 | |  |
| Temazepam | 301.157 | 283.200 | 81.00 | 17.00 | 4.00 | |  |
| Clobazam 1 | 301.166 | 259.100 | 91.00 | 27.00 | 4.00 | |  |
| Clobazam | 301.166 | 224.100 | 91.00 | 45.00 | 4.00 | |  |
| Zolpidem 1 | 308.283 | 235.100 | 96.00 | 45.00 | 4.00 | |  |
| Zolpidem | 308.283 | 263.100 | 96.00 | 29.00 | 4.00 | |  |
| Alprazolam 1 | 309.187 | 281.300 | 101.00 | 37.00 | 4.00 | |  |
| Alprazolam | 309.187 | 205.200 | 101.00 | 55.00 | 4.00 | |  |
| Flunitrazepam 1 | 314.212 | 268.300 | 96.00 | 37.00 | 4.00 | |  |
| Flunitrazepam | 314.212 | 239.100 | 96.00 | 49.00 | 4.00 | |  |
| Bromazepam 1 | 316.100 | 182.300 | 91.00 | 45.00 | 4.00 | |  |
| Bromazepam | 316.100 | 209.100 | 91.00 | 37.00 | 4.00 | |  |
| Clonazepam 1 | 316.156 | 270.200 | 96.00 | 35.00 | 4.00 | |  |
| Clonazepam | 316.156 | 214.200 | 96.00 | 53.00 | 4.00 | |  |
| Lorazepam 1 | 321.049 | 275.100 | 81.00 | 25.00 | 4.00 | |  |
| Lorazepam | 321.049 | 303.100 | 81.00 | 19.00 | 4.00 | |  |
| Alprazolam OH 1 | 325.170 | 297.200 | 111.00 | 31.00 | 4.00 | |  |
| Alprazolam OH | 325.170 | 216.200 | 111.00 | 55.00 | 4.00 | |  |
| Prazepam 1 | 325.208 | 271.200 | 91.00 | 25.00 | 4.00 | |  |
| Prazepam | 325.208 | 140.100 | 91.00 | 45.00 | 4.00 | |  |
| Midazolam 1 | 326.205 | 291.200 | 101.00 | 39.00 | 4.00 | |  |
| Midazolam | 326.205 | 249.200 | 101.00 | 51.00 | 4.00 | |  |
| Flunitrazepam OH | 330.191 | 284.200 | 76.00 | 23.00 | 4.00 | |  |
| Flunitrazepam OH 1 | 330.191 | 238.300 | 76.00 | 45.00 | 4.00 | |  |
| Lormetazepam 1 | 335.089 | 289.100 | 81.00 | 27.00 | 4.00 | |  |
| Lormetazepam | 335.089 | 317.200 | 81.00 | 17.00 | 4.00 | |  |
| Midazolam OH 1 | 342.167 | 324.000 | 91.00 | 25.00 | 4.00 | |  |
| Midazolam OH | 342.167 | 203.000 | 91.00 | 37.00 | 4.00 | |  |
| Triazolam 1 | 343.161 | 239.200 | 106.00 | 55.00 | 4.00 | |  |
| Triazolam | 343.161 | 308.200 | 106.00 | 29.00 | 4.00 | |  |
| Loflazepate 1 | 361.155 | 259.000 | 91.00 | 43.00 | 4.00 | |  |
| Loflazepate | 361.155 | 287.100 | 91.00 | 27.00 | 4.00 | |  |
| Zopicone DM 1 | 375.219 | 245.200 | 61.00 | 21.00 | 4.00 | |  |
| Zopicone DM | 375.219 | 111.900 | 61.00 | 79.00 | 4.00 | |  |
| Zopiclone 1 | 389.121 | 245.200 | 61.00 | 19.00 | 4.00 | |  |
| Zopiclone | 389.121 | 112.100 | 61.00 | 79.00 | 4.00 | |  |
| Loprazolam 1 | 465.106 | 408.000 | 106.00 | 29.00 | 4.00 | |  |
| Loprazolam | 465.106 | 111.100 | 106.00 | 33.00 | 4.00 | |  |

**Chromatographic separation:**

**For 1D liquid chromatography for AOC method:**

Time Module Events Parameter

1.00 Pumps Pump B Conc. 5

2.00 Pumps Pump B Conc. 40

4.00 Pumps Pump B Conc. 70

6.00 Pumps Pump B Conc. 100

7.00 Pumps Pump B Conc. 100

7.10 Pumps Pump B Conc. 5

10.00 System Controller Stop

Chromatographic column: biphenyl column (Phenomenex® Kinetex, 3 μm, 2.1 mm × 100 mm)

Mobile phases: 0.1% formic acid in water ; 0.1% formic acid in acetonitrile.

**For 1D liquid chromatography for sedatives and benzodiazepines**

Time Module Events Parameter

0.10 Pumps Pump B Conc. 10

1.80 Pumps Pump B Conc. 90

3.00 Pumps Pump B Conc. 90

3.10 Pumps Pump B Conc. 10

4.50 Pumps Pump B Conc. 10

4.51 System Controller Stop

Chromatographic column: biphenyl column (Phenomenex® Kinetex, 3 μm, 2.1 mm × 100 mm)

Mobile Phases: 10 mM ammonium formate and 1% acetic acid in water ; 0.1% formic acid in acetonitrile.

**For 2D liquid chromatography for cannabinoids**

Time Module Events Parameter

1.00 Pumps Pump B Conc. 20

1.00 Pumps Pump D Flow 0.0

1.00 Pumps Pump C Flow 1

1.10 Pumps Pump D Flow 1

1.10 Pumps Pump C Flow 0

6.50 Pumps Pump C Flow 0.0

6.50 Pumps Pump D Flow 1

6.60 Pumps Pump C Flow 0.0

6.60 Pumps Pump D Flow 1.5

7.50 Pumps Pump B Conc. 90

7.60 Pumps Pump B Conc. 100

8.50 Pumps Pump C Flow 0.0

8.50 Pumps Pump D Flow 1.5

8.50 Pumps Pump B Conc. 100

8.60 Pumps Pump C Flow 1

8.60 Pumps Pump D Flow 0

8.60 Pumps Pump B Conc. 20

10.00 System Controller Stop

Valco Valve Diverter

Total Time (min) Position

1 0.1 1D

2 1.0 2D

3 6.5 1D

Chromatographic column: XB C18 analytical column (Phenomenex® Kinetex, 5 μm, 2.1 mm × 50 mm)

Purification column: pentafluorophenyl (PFP) column (Phenomenex® Kinetex, 5 μm, 2.1 mm × 50 mm)

Mobile phases: 10 mM ammonium formate and 1% acetic acid in water ; 10 mM ammonium formate and 1% acetic acid in methanol.
