# Supplementary material for: Insights from a 6‐year hair drug analysis compendium in drug‐facilitated sexual assault cases
Source: J Forensic Sci. 2026 Apr 14;71(4):1783–92. doi: 10.1111/1556-4029.70331 (PMC13340955; doi:10.1111/1556-4029.70331)
Supplement: Supplementary file 2 — Table S2. [file JFO-71-1783-s001.docx]

TABLE S2 Validation data for the main drugs searched in hair sample.

|  | **LOD**  **(pg/mg)** | **LOQ**  **(pg/mg)** | **Linearity** | **Accuracy (n=6)** | | | **Precision (CV) (n=6)** | | |
| --- | --- | --- | --- | --- | --- | --- | --- | --- | --- |
| **Benzodiazepines** |  |  |  | QC  2 pg/mg | QC  8 pg/mg | QC  40  pg/mg | QC  2 pg/mg | QC  8 pg/mg | QC  40  pg/mg |
| 7-aminoclonazepam | 2.5 | 5 | 5-500 | 5,5% | 1,7% | -1,8% | 10,1% | 8,4% | 9,7% |
| 7-aminoflunitrazepam | 2.5 | 5 | 5-500 | 5,2% | -4,7% | -4,1% | 11,4% | 12,2% | 11,3% |
| alprazolam | 0.5 | 1 | 1-500 | 3,8% | 2,2% | 2,9% | 14,8% | 9,1% | 4,6% |
| bromazepam | 2.5 | 5 | 5-500 | 2,1% | 1,8% | 8,0% | 13,5% | 7,8% | 11,1% |
| clobazam | 1 | 2.5 | 2.5-500 | 4,7% | -4,5% | 0,5% | 9,3% | 6,9% | 7,2% |
| clonazepam | 1 | 2.5 | 2.5-500 | -2,1% | 2,2% | 4,7% | 10,5% | 9,8% | 5,7% |
| desmethylclobazam | 1 | 2.5 | 2.5-500 | 11,3% | -6,6% | -5,4% | 5,6% | 11,5% | 6,2% |
| desmethylflunitrazepam | 1 | 2.5 | 2.5-500 | 3,2% | -4,2% | 5,0% | 13,0% | 4,4% | 7,2% |
| diazepam | 1 | 2.5 | 2.5-500 | -8,8% | -14,1% | -4,1% | 12,7% | 8,8 % | 5,8% |
| estazolam | 1 | 2.5 | 2.5-500 | 4,7% | -3,8% | 0,4% | 14,0% | 5,3% | 5,8% |
| flunitrazepam | 0.5 | 1 | 1-500 | 4,5% | -1,1% | 4,5% | 7,0% | 8,5% | 5,9% |
| hydroxyalprazolam | 1 | 2.5 | 2.5-500 | 9,3% | 5,8% | 4,6% | 10,9% | 6,7% | 10,3% |
| hydroxyflunitrazepam | 2.5 | 5 | 5-500 | 2,4% | 2,7% | 10,4% | 7,8% | 9,6% | 7,8% |
| hydroxymidazolam | 2.5 | 5 | 5-500 | -1,2% | -6,4% | 5,5% | 9,9% | 6,0% | 4,6% |
| loflazepate | 1 | 2.5 | 2.5-500 | -4,5% | -8,2% | -2,0% | 10,9% | 11,5% | 6,0% |
| loprazolam | 1 | 2.5 | 2.5-500 | -1,7% | -3,6% | 3,0% | 9,2% | 12,0% | 10,9% |
| lorazepam | 5 | 10 | 10-500 | 11,0% | -3,5% | 4,5% | 7,4% | 2,5% | 8,5% |
| lormetazepam | 2.5 | 5 | 5-500 | 1,5% | -2,3% | -1,9% | 8,9% | 9,1% | 11,8% |
| midazolam | 1 | 2.5 | 2.5-500 | 7,8% | -0,6% | 7,4% | 6,3% | 10,2% | 9,9% |
| nitrazepam | 1 | 2.5 | 2.5-500 | 0,9% | 3,1% | 7,0% | 13,7% | 7,5% | 3,8% |
| nordiazepam | 2.5 | 5 | 5-500 | -7,4% | -15,0% | -4,2% | 11,1% | 7,2% | 10,3% |
| oxazepam | 5 | 10 | 10-500 | 7,3% | 2,0% | 0,0% | 9,6% | 7,4% | 4,7% |
| prazepam | 1 | 2.5 | 2.5-500 | 1,5% | -11,9% | 4,2% | 10,3% | 14,1% | 9,2% |
| temazepam | 1 | 2.5 | 2.5-500 | -0,2% | -8,3% | -4,5% | 8,2% | 7,9% | 2,0% |
| tetrazepam | 2.5 | 5 | 5-500 | 2,4% | 3,0% | 2,7% | 8,4% | 12,7% | 10,4% |
| triazolam | 1 | 2.5 | 2.5-500 | 3,4% | 0,9% | 4,2% | 14,2% | 13,4% | 9,1% |
| zolpidem | 0.5 | 1 | 1-500 | 5,6% | 1,9% | 8,4% | 8,4% | 8,9% | 7,1% |
| zopiclone | 0.5 | 1 | 1-500 | -1,9% | 0,3% | 3,3% | 14,5% | 8,3% | 10,7% |
| **Sedatives** |  |  |  | QC  2 pg/mg | QC  8 pg/mg | QC  40  pg/mg | QC  2 pg/mg | QC  8 pg/mg | QC  40  pg/mg |
| alimemazine | 0.5 | 1 | 1-500 | 2,8% | -1,9% | 3,8% | 11,6% | 7,7% | 8,8% |
| amitriptyline | 0.5 | 1 | 1-500 | 8,2% | 12,7% | 10,3% | 4,5% | 1,7% | 1,5% |
| amoxapine | 0.5 | 1 | 1-500 | 1,7% | 5,8% | 4,4% | 6,9% | 8,0% | 5,6% |
| atropine | 2.5 | 5 | 5-500 | 4,5% | -3,9% | 3,0% | 14,4% | 13,5% | 10,9% |
| brompheniramine | 1 | 2.5 | 2.5-500 | 2,7% | 1,5% | 4,8% | 12,3% | 14,7% | 8,0% |
| cetirizine | 1 | 2.5 | 2.5-500 | -1,4% | 3,9% | -1,8% | 9,0% | 11,5% | 9,1% |
| chlorpheniramine | 1 | 2.5 | 2.5-500 | 4,2% | 2,5% | -3,7% | 14,0% | 13,2% | 11,6% |
| chlorpromazine | 1 | 2.5 | 2.5-500 | 7,8% | -5,5% | -0,6% | 9,3% | 7,7% | 6,6% |
| clozapine | 1 | 2.5 | 2.5-500 | -0,3% | 2,4% | -1,3% | 18,3% | 10,5% | 8,1% |
| cyamemazine | 0.5 | 1 | 1-500 | 2,7% | -3,2% | 2,4% | 10,8% | 12,0% | 10,5% |
| diphenydramine | 1 | 2.5 | 2.5-500 | -0,4% | 2,2% | 1,5% | 10,3% | 9,4% | 13,7% |
| doxylamine | 0.5 | 1 | 1-500 | 3,0% | 4,8% | 0,1% | 10,3% | 12,9% | 12,8% |
| haloperidol | 0.5 | 1 | 1-500 | 4,9% | -3,8% | 2,5% | 9,2% | 11,2% | 8,5% |
| hydroxyzine | 0.5 | 1 | 1-500 | 6,7% | 0,8% | 3,9% | 12,2% | 6,4% | 6,7% |
| ketamine | 5 | 10 | 10-500 | -0,3% | -2,4% | 4,3% | 10,7% | 14,8% | 14,5% |
| levomepromazine | 0.5 | 1 | 1-500 | 0,7% | -10,6% | 1,0% | 10,7% | 8,6% | 9,3% |
| loratadine | 2.5 | 5 | 5-500 | 2,8% | -5,6% | -0,6% | 14,9% | 5,7% | 13,7% |
| loxapine | 0.5 | 1 | 1-500 | 10,7% | 9,9% | 1,8% | 7,4% | 9,5% | 5,2% |
| mirtazapine | 0.5 | 0.5 | 0.5-500 | - 1,5 % | 5,2 % | 2.3 % | 10,5% | 8,3% | 5,7% |
| niaprazine | 0.5 | 1 | 1-500 | 5,5% | -1,3% | 5,7% | 12,1% | 12,6% | 9,7% |
| norketamine | 5 | 10 | 10-500 | 0,4% | -2,0% | 1,7% | 14,7% | 8,8% | 7,6% |
| normirtazapine | 1 | 1 | 1-500 | 1,9% | - 5,8% | 4,3% | 9,8% | 7,1% | 4,3% |
| o-desmethyltramadol | 1 | 2.5 | 2.5-500 | 10,9% | 6,4% | 4,1% | 5,1% | 5,2% | 5,4% |
| paliperidone | 0.5 | 1 | 1-500 | 4,6% | 0,6% | 1,1% | 13,2% | 9,4% | 13,0% |
| paroxetine | 2.5 | 5 | 5-500 | 6,2% | 1,3% | 8,3% | 5,5% | 7,5% | 13,4% |
| pheniramine | 1 | 2.5 | 2.5-500 | 0,2% | -3,4% | 4,6% | 8,8% | 12,6% | 10,7% |
| promethazine | 2.5 | 5 | 5-500 | 1,6% | -6,8% | 1,7% | 14,9% | 11,2% | 12,2% |
| risperidone | 0.5 | 1 | 1-500 | 5,7% | -3,5% | 5,7% | 13,7% | 8,9% | 12,8% |
| scopolamine | 1 | 2.5 | 2.5-500 | 3,8% | -3,3% | -0,2% | 10,1% | 10,6% | 11,3% |
| tiapride | 1 | 1 | 1-500 | 9,9% | 6,9% | - 4,6% | 12,2% | 7,8% | 5,6% |
| tramadol | 1 | 2.5 | 2.5-500 | 10,1% | 4,2% | 1,2% | 2,0% | 5,7% | 3,8% |
| trihehyphenidyl | 0.5 | 1 | 1-500 | 5,1% | -6,7% | 4,7% | 14,3% | 4,9% | 6,9% |
| tropatepine | 1 | 2.5 | 2.5-500 | 3,1% | -2,8% | 3,2% | 13,2% | 8,5% | 12,4% |
| **Narcotics** |  |  |  | QC  75 pg/mg | QC  125 pg/mg | QC  1250  pg/mg | QC  75 pg/mg | QC  125 pg/mg | QC  1250  pg/mg |
| amphetamine | 10 | 10 | 10-5000 | 4.0 % | 9.7 % | -0.2 % | 9,3 % | 13.6 % | 11.6 % |
| methamphetamine | 10 | 10 | 10-5000 | -3,7 % | -1.4 % | -9.6 % | 10,9 % | 12.3 % | 5.4 % |
| MDMA | 10 | 10 | 10-5000 | -3.7 % | 0.5 % | -13.8 % | 9,7 % | 9.0 % | 13.3 % |
| MDA | 10 | 10 | 10-5000 | 0.9 % | 2.4 % | -0.3 % | 8,2 % | 10.4 % | 5.7 % |
| MDEA | 10 | 10 | 10-5000 | -1.6 % | 4.0 % | -10.9 % | 2,3 % | 14.0 % | 7.2 % |
| cocaine | 10 | 10 | 10-5000 | 0.4 % | 5.9 % | 3.0 % | 11,1 % | 9.1 % | 4.7 % |
| benzoylecgonine | 10 | 10 | 10-5000 | -1.7 % | 2.0 % | -14.7 % | 7,9 % | 9.4 % | 7.0 % |
| ecgonine methyl ester | 10 | 10 | 10-5000 | -2.4 % | 3.0 % | -4.2 % | 10,4 % | 7.8 % | 8.1 % |
| cocaethylene | 10 | 10 | 10-5000 | -1.7 % | 1.6 % | -5.3 % | 12,8 % | 10.5 % | 8.1 % |
| norcocaine | 10 | 10 | 10-5000 | N/A | N/A | N/A | N/A | N/A | N/A |
| morphine | 10 | 10 | 10-5000 | -7.9 % | -2.5 % | -3.4 % | 11,1 % | 7.9 % | 2.9 % |
| 6-MAM | 10 | 10 | 10-5000 | -6.3 % | 3.7 % | 0.3 % | 12,3 % | 10.9 % | 6.1 % |
| codeine | 10 | 10 | 10-5000 | -1.6 % | 3.7 % | 1.8 % | 9.8 % | 9.3 % | 6.3 % |
| ethylmorphine | 10 | 10 | 10-5000 | -8.0 % | -1.6 % | -2.1 % | 11.1 % | 7.9 % | 7.8 % |
| dihydrocodéine | 10 | 10 | 10-5000 | -3.9 % | 6.0 % | -2.2 % | 10.9 % | 7.3 % | 6.0 % |
| pholcodine | 10 | 10 | 10-5000 | -7.6 % | 2.3 % | -1.6 % | 11.3 % | 9.7 % | 13.5 % |
| oxycodone | 10 | 10 | 10-5000 | -2.0 % | 3.1 % | 7.5 % | 9.8 % | 8.3 % | 10.2 % |
| methadone | 10 | 10 | 10-2000 | N/A | N/A | N/A | N/A | N/A | N/A |
| EDDP | 10 | 10 | 10-2000 | N/A | N/A | N/A | N/A | N/A | N/A |
| Cannabinoids |  |  |  | QC  3 pg/mg | QC  30 pg/mg | QC  300  pg/mg | QC  3 pg/mg | QC  30 pg/mg | QC  300  pg/mg |
| THC | 1 | 5 | 5-500 | -8,7% | -3,2% | 1,9% | 8,9% | 8,5% | 4,1% |
| CBD | 10 | 10 | 10-2000 | 8,7% | 3,8% | 1,3% | 7,4% | 5,7% | 8,5% |
| THC-COOH | 1 | 1 | 1-500 | -3,7% | 1,8% | 0,4% | 3,7% | 10,3% | 9,1% |
| CBN | 30 | 30 | N/A | N/A | N/A | N/A | N/A | N/A | N/A |
| Others |  |  |  |  |  |  |  |  |  |
| LSD | 1 | 1 | N/A | N/A | N/A | N/A | N/A | N/A | N/A |

**Lower limit of detection : LOD**

**Lower limit of quantification : LOQ**
